# 

**Published:** 1883-11

**Authors:** 


Chicago Medical Press Association.

Aug. 2D

1875.

CHICAGO:

*PUBLISHED BY THE CHICAGO MEDICAL PRESS ASSOCIATION*

188 Clark Street.

**☞ Read the Notice of this Journal among Advertisements**

